# Edible Biopolymers-Based Materials for Food Applications—The Eco Alternative to Conventional Synthetic Packaging

**DOI:** 10.3390/polym13213779

**Published:** 2021-10-31

**Authors:** Roxana Gheorghita Puscaselu, Irina Besliu, Gheorghe Gutt

**Affiliations:** 1 Faculty of Medicine and Biological Sciences, Stefan cel Mare University of Suceava, 720229 Suceava, Romania; 2 Integrated Center for Research, Development and Innovation in Advanced Materials, Nanotechnologies, and Distributed Systems for Fabrication and Control, Stefan cel Mare University of Suceava, 720229 Suceava, Romania; 3 Faculty of Mechanical Engineering, Automotive and Robotics, Stefan cel Mare University of Suceava, 720229 Suceava, Romania; besliu.irina@usm.ro; 4 Faculty of Food Engineering, Stefan cel Mare University of Suceava, 720229 Suceava, Romania; g.gutt@fia.usv.ro

**Keywords:** agar, alginate, food supplements, hydrosolubility

## Abstract

The problem of waste generated by packaging obtained from conventional synthetic materials, often multilayer, has become more and more pressing with increasing consumption. In this context, nature and humanity have suffered the most. In order to address this phenomenon, global and European organizations have launched and promoted programs and strategies. Replacing petroleum-based packaging with biopolymer packaging has proven to be a real alternative. Thus, the substitution of plastics with biodegradable, non-toxic, edible materials, which can be obtained from marine or agro-industrial waste, is of interest. In the present study, we aimed to develop natural edible materials, obtained entirely from biopolymers such as agar and sodium alginate and plasticized with glycerol and water. Designed to be used for food and food supplements packaging, they can be completely solubilized before consumption. The films were developed through a casting method and were tested in order to identify the physical, optical, and solubility properties. According to the results, the most suitable composition for use as a hydrosoluble packaging material contains agar:alginate:glycerol in a 2:1:1 ratio. The microstructure indicates a homogeneous film, with low roughness values (Rz = 12.65 ± 1.12 µm), high luminosity (92.63), above-average transmittance (T = 51.70%), and low opacity (6.30 A* mm^−1^). The obtained results are of interest and highlight the possibility of substituting intensely polluting materials with those based on biopolymers.

## 1. Introduction

The packaging for the food and medical industry involves a number of multidisciplinary areas, such as engineering, chemistry, biotechnology, and microbiology. The efficiency of a good packaging material is influenced by its ability to preserve the nutritional and sensory qualities of the product contained, to preserve the consumption and microbiologically safety, and, last but not least, to facilitate the promotion and sale of the product. The main feature of a good packaging material is to maintain the quality and shelf life of the product from manufacturing to the consumer. The shelf life represents the time from product development to consumption, the period in which the product retains its quality under certain storage conditions (according to the manufacturers’ specifications). Therefore, the shelf life of food is highly associated with the inherent characteristics of the food and the environmental conditions exposed during transport and storage and, most importantly, with the quality of the packaging system used [1].

A good packaging material should (i) protect the packaged product from physical and mechanical factors; (ii) prevent damage to the product due to the actions of the natural environment: light, radiation, heat, humidity; (iii) prevent the incidence and proliferation of microorganisms; (iv) ensure stability against oxidative processes; (v) prevent unpleasant odors and maintain taste and color; (vi) allow and ensure the transfer of nutrients; and (vii) function as a sensor carrier [2].

A successful alternative could be biopolymer-based packaging materials. If at first biopolymers were used in the food industry as additives and ingredients, studies have shown new innovative properties and possible new applications. Most biopolymers used to make edible films are those based on proteins, lipids, and polysaccharides. They can be obtained by extraction from seaweed or waste and by-products from the agri-food industry, with economic and environmental benefits. These materials are non-toxic, non-allergenic and completely biodegradable and compostable, generating water, CO_2_, and biomass under the action of physical factors and decomposing microorganisms [3]. Due to the advantages in use, the use of biopolymers has expanded into other industries, such as the pharmaceutical and biomedical industries. In this way, they contributed to the development of new materials, such as scaffolds, wounds, tissue engineering, etc., and in the pharmaceutical industry especially for microencapsulation of drugs and probiotics [4]. The continuous testing of biopolymers has facilitated the identification of new techniques for obtaining materials, with new characteristics and contributions to the development of innovative products.

An edible film is a thin foil used for packaging products [5]. A coating is a layer applied directly to the surface of the product, and maintained until completely dry. Usually, the coatings are applied to perishable fruits and vegetables. For microbiological safety, they may contain organic acids, bacteriocins, or natural extracts [6].

A film with good properties must have some characteristics, such as (a) protecting the product it contains, (b) very good sensory qualities, barrier properties, or mechanical performance, (c) microbial stability and no toxins, (d) safe, and (e) easy to obtain, with low cost, and easy to handle and use [7]. Moreover, due to its composition, development, and use, various beneficial substances, such as powders from natural products [8,9,10], essential oils [11,12], dyes [13], and flavorings [14], can be incorporated into the film-forming solutions. These substances can be consumed together with the packaging material and can improve the characteristics of the final product [15]. Because the performance of these films cannot reach that of plastics, which are more resistant, especially in terms of mechanical and barrier properties, new techniques for obtaining biopolymeric materials have been developed. Layer-by-layer self-assembly is a common technique in interface science to produce biobased-films and a method widely used today for the development of natural materials, with properties similar to synthetic ones. The method involves alternating positively and negatively charged layers and thus high-performance smart materials can be obtained [16]. The development of composite materials, obtained by combining two or more biopolymers with a synergistic effect, is another method of improving the existing properties. In addition to these favorable properties, this type of material is one that generates zero waste. This is a very important aspect if we take into account that, at present, pollution due to plastic waste has reached alarming levels, and most of waste it is thrown into the seas and oceans [17]. Additionally, a topical problem is the transformation of plastic into microplastic, which is toxic to the body [18]. If 20 years ago, the percentage of plastic waste did not exceed 100 million tons/m^2^, then in 2018, the value reached 330 tons/m^2^, and so in 2020, it reached 400 t/m^2^. The repercussions have a negative impact on human and animal health, causing soil, water, and air pollution [19]. The European Union is making considerable efforts to eradicate these practices, and countries such as Sweden, Germany, Austria, Denmark, and the Netherlands are pursuing an 80–100% plastic recovery policy [20]. One way might be to approach a circular economy. The world economy is currently estimated to be less than 10% circular [21]. A green strategy starts from the home of each inhabitant of the planet that produces garbage, and is connected to small, medium, large and very large factories [22]. Such a strategy should point out that the degree of global urbanization will increase from 54% (2020) to 75% (2050) [23]. The circular economy uses existing materials, to which it assigns new values, and avoids overexploitation [24]. The current trend is to use biodegradable, compostable, or renewable materials, or those which ultimately lead to zero waste.

This paper aims to develop and test completely natural and edible foils that can be used as packaging material for powdered food supplements. Based on the use of biopolymers such as agar and sodium alginate, but also glycerol for plasticization, the materials obtained were tested to identify specific characteristics. Thus, the physical and optical properties, such as microstructure, microtopography, and the uniformity and regularity of surfaces, but also the thickness, the retraction ratio, the color parameters, the transmittance, and the opacity were evaluated. The determinations were supplemented with the evaluation of solubility (water solubility, swelling ratio, moisture content, water activity index), and microbiological characteristics.

The food supplement industry is constantly growing, especially due to changing habits and refining consumer tastes. Usually, this type of packaging is multilayer, difficult to sort and impossible to recycle. The materials developed represent a viable option for conventional packaging.

## 2. Materials and Methods

All the substances used for the development of the films were purchased from the company Sigma Aldrich (Romanian branch from Bucharest).

To obtain the film-forming solutions, a volume of 150 mL of water and different amounts of biopolymers and plasticizer were used, with a total mass of 4 g. The different masses of plasticizer were 0.5, 0.75, and 1 g, and the mass of biopolymers varied in the range of 0–3 g, as shown in Table 1.

**Figure 1:**
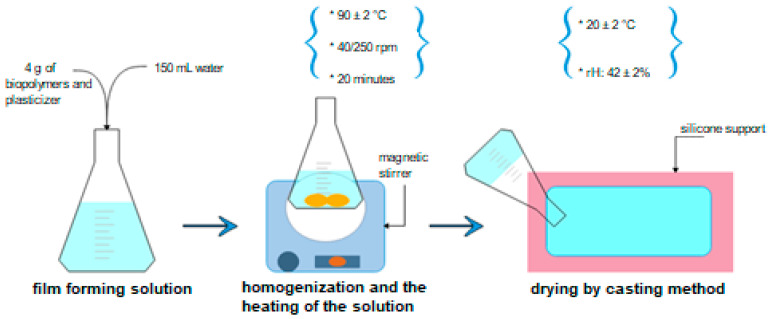
Graphic representation of films’ development.

The films have been tested to identify specific characteristics and the possibility of use for the packaging of food supplements. To obtain homogeneous solutions, they were mixed using a homogenizer with heating, as follows: homogenization and maintenance for 20 min at 90 ± 2 °C and 400 rpm for the first 10 min or until the solution is clarified, followed by 250 rpm until at the end of the allotted time (Figure 1). Subsequently, they were poured onto the silicone foil and maintained until completely dry.

- Evaluation of physical and optical properties

To evaluate the microstructure and microtopography of the samples obtained, the films were tested using the Mahr confocal microscope (Marf Surf). These properties, in conjunction with the other tests, are important for any newly developed material, particularly for materials that will subsequently be used in packaging and must meet a number of conditions, including maintaining the quality of the product, from manufacturer to the final consumer, especially because the visual aspect is important and is used as a marketing strategy. In this sense, we made a series of determinations.

- The evaluation of thickness and retraction ratio

Thickness is an important feature for a film that will be used as packaging material for various products. The Mitutoyo micrometer was used to measure the thickness in at least 5 different areas of the film; the noted result represents their arithmetic mean. 

The retraction ratio was calculated according to the following formula:

$$\mathrm{Retraction}\;\mathrm{ratio},\;\left(\%\right)=\frac{\left(\mathrm{initial}\;\mathrm{film}\;\mathrm{thickness}-\mathrm{final}\;\mathrm{film}\;\mathrm{thickness}\right)}{\mathrm{initial}\;\mathrm{film}\;\mathrm{thickness}}*100$$

- The determination of color, transmittance, and opacity

The color of the samples was evaluated using the Konika Minolta CR 400 colorimeter (Konika Minolta, Tokyo, Japan), and was observed on different areas of the film surface. The method evaluates the color of the products using the CIELAB system, and the determinations aimed at identifying the luminosity (L*) and the coordinates a* (green-red) and b* (blue-yellow). In the CIELAB system, the luminosity has values between 0 (black range) up to 100 (absolute white). In order to establish the results, five readings were performed, and the final value represents their arithmetic mean. Both the transmittance and the opacity of the films were evaluated using the Ocean Optics HR 4000 CG-UV-NIR spectrophotometer (Ocean Optics, Douglas, AZ, USA). Thus, 1 cm × 3 cm specimens were introduced into the spectrophotometric tank and the values indicated at the wavelength of 660 nm (for transmittance) and 600 nm (for opacity) were read.

Opacity (O) is calculated according to the formula:

$$O,\;\left(\frac{A}{\mathrm{mm}}\right)=\frac{A}{T}$$

where A is absorbance and T is thickness (mm).

The evaluation of these characteristics is important for maintaining the quality of packaged products (depending on the content, there are products that degrade under the action of light rays) and in the marketing strategy of the product, as colored packaging is often more interesting than colorless [25].

- Solubility evaluation

The solubility of films is an important feature, especially when they are intended for use as packaging materials, as their physical properties can be affected by environmental humidity [26]. As the films developed are intended to be used for the packaging of food, which requires solubilization, and consumed with them, the assessment of solubility is of the utmost interest. Moreover, knowing these characteristics, it can be indicated the type of products which can be packaged in various materials. Depending on the moisture content and the final values of the hydration capacity and swelling ratio index, the path of the foils can be traced as packaging materials for various products.

The standardized method according to STAS 90–88 was used to determine the humidity. Thus, the film samples (3 cm × 3 cm) were weighed and maintained for 24 h at 110 °C, then reweighed. The values obtained were used in the following formula:

$$\mathrm{MC},\;\left(\%\right)=\frac{W0-\;W1}{W0}\;*100$$

where W_0_ represents the mass of the sample before drying (g) and W1 is its mass after drying (g).

To determine films’ water solubility, specimens similar to those used to determine moisture content (3 cm × 3 cm) were weighed and kept for 8 h in containers with 50 mL of water. After 8 h, the samples were dried using a hot air oven (Memmert Schwabach, Germany) for 24 h at 110 °C. The result was calculated according to the following formula:

$$\mathrm{WS},\;\left(\%\right)=\frac{W0-\;W1}{W0}\;*100$$

where W_0_ is the mass of the sample before immersion in water (g) and W_1_ is the mass of the sample (g) after 24 h.

The hydration capacity of the foils was determined by maintaining a sample (3 × 3 cm) for 30 s to 20 min in water with a temperature of 22 ± 2 °C. After the predetermined time, the sample was lightly taped with filter paper in order to remove the excess water and then reweighed.

The final value of swelling capacity (SR) was calculated with the formula:

$$\mathrm{SR},\;\left(\%\right)=\frac{\mathrm{Wt}\;-\;W0}{W0}\;*100$$

where W_0_ represent the initial mass (g) and W_t_ is the sample mass after the time t, (g).

The water activity index (a_w_) is an important parameter, especially if we take into account that its low value, in the case of tested samples, also highlights the ability to provide protection against microbial contamination. The water activity index was determined using Aqua Lab equipment (ICT International, Armidale, NSW 2350, Australia), and the determinations were performed at a temperature of 23 ± 2 °C. The value noted represent the sum of least five determinations made in different areas of the film surface.

- The microbiological assessments

As the films developed are intended for the packaging of food supplements and consumed with them, it is imperative to be safe from this point of view. Thus, the incidence of microorganisms was tested using Compact Dry TC/CF/ETC/ETB/EC/XSA/LM/YM dehydrated specific culture media plates (NISSUI Pharma).

In this regard, the total count (TC), the incidence of coliform bacteria (CF), *Enterococcus* (ETC), enterobacteria (ETB), *Escherichia coli* (EC), *Staphylococcus aureus* (XSA), *Listeria monocytogenes* (LM), and yeasts and molds (YM) were tested.

For these determinations, saline was used to hydrate the medium. Thus, 1 g of sample was homogenized with 9 mL of saline. From the solution, 1 mL was poured onto the culture media. After hydration, the plates with culture media were maintained at 72 °C for 24–36 h for TC/CF/ETC/ETB/EC/XSA/LM/YM determinations and 72 h for plates with specific yeast and mold culture media. In order to obtain precise results, for each sample tested, we performed three dilutions.

- Statistical analysis

The purpose of the statistical determinations was to optimize the composition for the development of materials with improved properties. For this purpose, the Design Expert 12 (State Ease) program was used, generating SRM responses. Significant differences were evaluated by carrying out one-way analysis of variance (ANOVA) and Tukey’s test at *p* < 0.05. Data analysis was performed using MiniTAB statistics software (MiniTAB Ltd., Coventry, UK).

## 3. Results

For drying, the film-forming solutions were maintained for about 48 h at a temperature of 24 ± 0.5 °C and 39 ± 1% relative humidity. The composition of the films and their physical characteristics can be found in Table 1. In order to establish the conditions for maintaining the solutions until complete drying and film development, as well as to establish the characteristics necessary for future industrial use, the adhesiveness, the appearance of the films, and possible mixtures with other biopolymers were evaluated. The results are useful for improving the characteristics and obtaining industrial performances. According to Table 1, the compositions most suitable for packaging materials are those of samples B2, B3, B10, and B13–B16. Their common feature is the increase in the plasticizer content in the composition. The exception is sample B3, which, however, contains a higher amount of sodium alginate. Other studies have reported the ability of agar to form hard films and alginate to facilitate the development of glossy and flexible films [27]. The films were kept at constant temperature and rH. Samples B1–B16 showed different characteristics and behavior (Table 2), a beneficial aspect if we take into account that they can be used later for packaging products with different properties.

The difference between B3 and B14 is represented by the decrease in the agar mass and the increase in the plasticizer mass. According to the results (Table 3), the best characteristics for use as packaging materials are those of the last sample.

**Figure 2:**
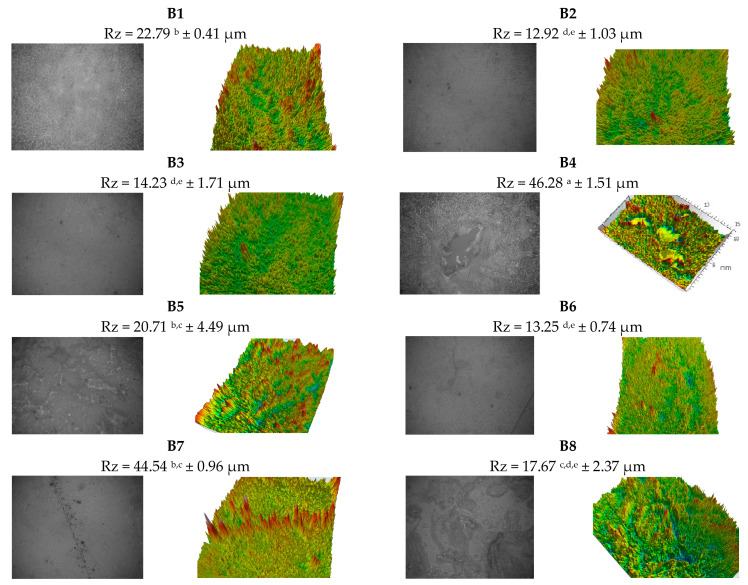

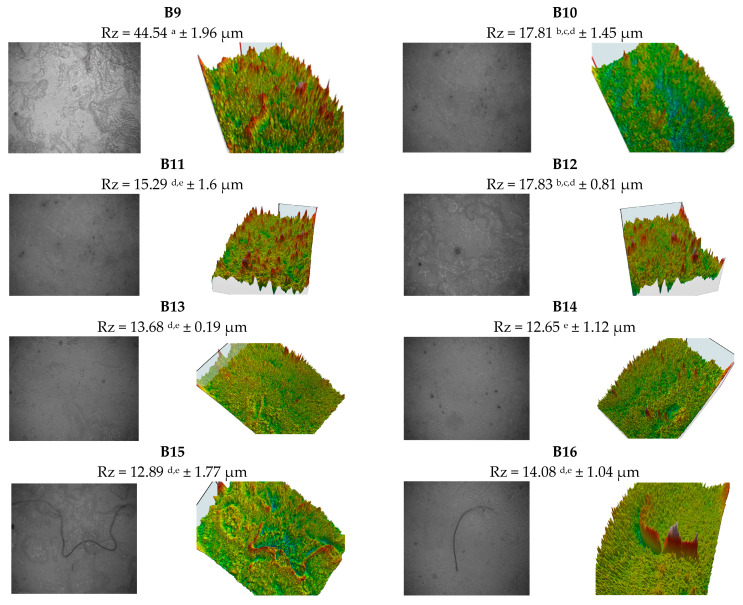
SEM images and microtopographies of the tested foils. Values (a–e) followed by the same letter are not significantly different (α: 0.05).

According to the images in Figure 2, the samples with higher content of plasticizer in the composition (B13–B16) show a more homogeneous and compact structure, but also a lower roughness. The reduced roughness may be due to the glycerol molecules which, due to the reduced formations, have the possibility to integrate much more easily in the film matrix. Reduced roughness is observed in the samples with higher agar content in the composition, as evidenced by the low standard deviations, with values of 0.19 (B13), 0.41 (B1), 0.74 (B6), 0.81 (B12), and 0.96 (B7).

The retraction ratio is an important and useful determination for the manufacturer, because, depending on the thickness of the film-forming solution and the value of the retraction ratio, the final thickness of the foil can be determined in advance and accurately. In this way, the manufacturer has the ability to determine the thickness of the material obtained. As can be seen from Table 2, the lowest values of the retraction ratio are observed in the samples with the highest values of thickness, namely B1 and B4. The retraction ratio is inversely proportional to the thickness; thus, where the thickness has low values, the shrinkage ratio has high values (B5, B10, B2, B13). 

The lowest thickness can be seen in sample B5, with an equal content of biopolymers in composites, although the amount of plasticizer is small. Studies have presented that a high amount of plasticizer in the composition leads to thin and flexible films [28]. According to the microstructure presented in the images in Figure 2, the plasticizer used, which was glycerol in this case, is compatible with the biopolymers used. When the plasticizer is not used in the right amount or is not compatible with the other substances, visible visual defects can be observed: cracks, the tendency to break, white color, and high opacity, properties that can be attributed to phase separations [29]. The values obtained after evaluating the moisture content, water solubility, and water activity index are presented in Table 4.

The solubility of new materials is of interest due to their subsequent applicability. As the study aimed to develop films that can be used for food packaging and food supplements, the films can be consumed with the product when it is immersed in liquids with temperatures above 45 °C.

The high agar content of the composition facilitated the decrease in luminosity (the lowest in the case of sample B1-86,221, where the mass of agar is 3 g). The highest value of luminosity—92,772—was found in sample B10, which, although there are no large variations between the masses of the two biopolymers, contains a higher amount of plasticizer in the composition (0.75 g).

**Figure 3:**
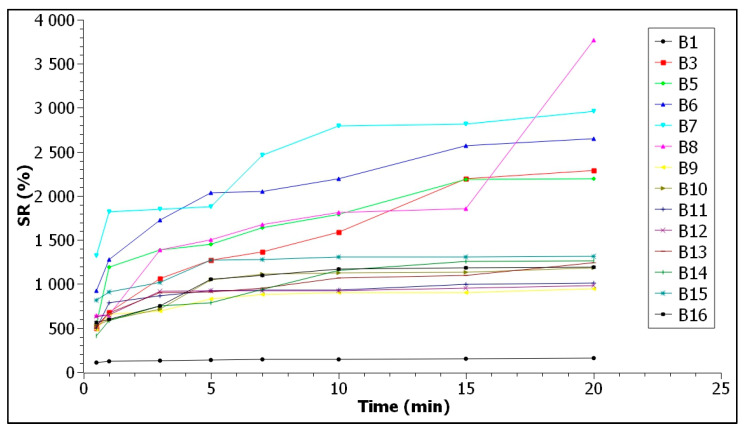
Swelling ratio of the films.

After testing the selling ratio capacity, according to the graph in Figure 3, samples B2 and B4 could not be evaluated, as they disintegrated in the first 30 s of water immersion. Sample B4 showed a tendency to flake off and disintegrated immediately. According to these determinations, these foils can be used for the packaging of products with low humidity (such as powders, tablets, instant drinks, powdered milk, etc., which require solubilization before consumption). 

The films B1, B3, B6, and B9 kept their shape very well, even 20 min after immersion in water. According to the graph, samples B1 and B9 had a low water retention capacity, demonstrated by the low values of the swelling ratio of 157.43% and 949%, respectively, after 20 days of immersion. According to Table 1, film B1 has only agar and glycerol in its composition, unlike film B9, which contains equal masses of biopolymers, but less glycerol. Film B3, with a higher agar content in composition, and film B6, with more sodium alginate, although they had high values of rehydration capacity (2292.27% and 2650.28%, respectively), kept their shape and did not solubilize. These materials can be used for packaging products with higher moisture content, such as fruits and vegetables, meat and meat products, or other products with similar characteristics. Samples B10, B11, and B12, although they had low values of hydration capacity (1183.45%, 1008.05%, and 980%) and did not disintegrate after 20 min of immersion, did not keep their uniformity, like the other films.

**Figure 4:**
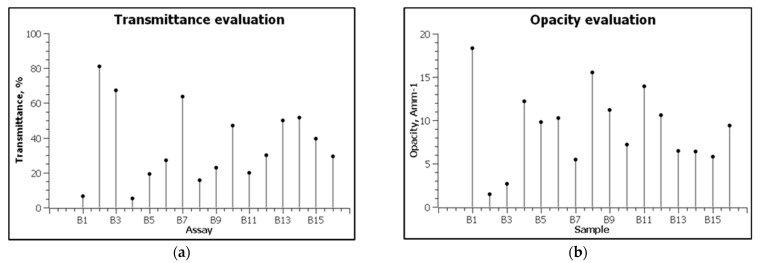
The transmittance (**a**) and opacity (**b**) of developed films.

The high alginate content resulted in films with high transmittance values (Figure 4). Thus, according to the obtained results, samples B2 (3 g alginate, *T*—81.10%) and B7 (2.5 g alginate, *T*—63.79%) showed higher transmittance as opposed to those with higher agar content. Exceptions are samples B3 and B14; although the amount of agar was higher, the films showed high values of the transmitter (67.17% and 51.70%, respectively). This behavior could be attributed to the higher mass of plasticizer in the film composition or due to the synergistic effect between the compounds used.

Conversely, samples with low values other than transmittance showed high opacity. For example, the opacity of sample B1, with 3 g of agar and without sodium alginate in the composition, was 18.33 A* mm^−1^, followed by sample B8, with 15.56 A* mm^−1^, and then B11, with 13.94 A* mm^−1^. According to the obtained results, it is possible to identify a well-established correlation between the composition of the films and the opacity, since the three samples with high values show different proportions of biopolymers.

**Figure 5:**
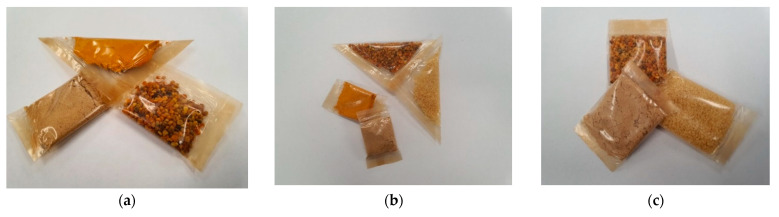
The applications of developed biopolymers-based materials: food supplements (ginger powder, turmeric, pollen, couscous) packed in B14 (**a**), B15 (**b**), and B16 (**c**) films.

The applicability of these foils is intensively studied, especially due to the common properties with those of conventional packaging materials. In the images in Figure 5, various applications of the new materials can be observed. Depending on the results obtained, samples B14, B15, and B16 were used. They can be intended for the packaging of food products and supplements that require solubilization in water with a temperature above 40 °C, before consumption. These packaging materials can be used for a wide range of foods, especially those with low moisture content. According to results presented in Table 4, the increase in the agar mass in the film composition facilitates the obtaining of biopolymer materials with low solubility, which can be used for the packaging of products with higher moisture content.

The evaluation of the microbiological characteristics highlighted the safety of ingestion of these films when they are consumed together with the packaged product. None of the microorganisms tested, such as coliforms, enterococci, enterobacteria, *Escherichia coli*, *Staphylococcus aureus*, *Listeria monocytogenes,* or yeasts and molds, developed on the culture medium. The incidence and proliferation of microorganisms was also hampered by the low values of the water activity index. Following the determinations, its values varied between 0.414 and 0.457, being small enough to prevent microbial contamination. This feature is extremely important because the preservation of nutritional and sensory characteristics, but also the preservation of the product’s dependence depends on the presence of spoilage microorganisms. Additionally, the incidence of pathogenic microorganisms endangers the health of the consumer, which is excluded in this case.

## 4. Statistical Analysis

**Figure 6:**
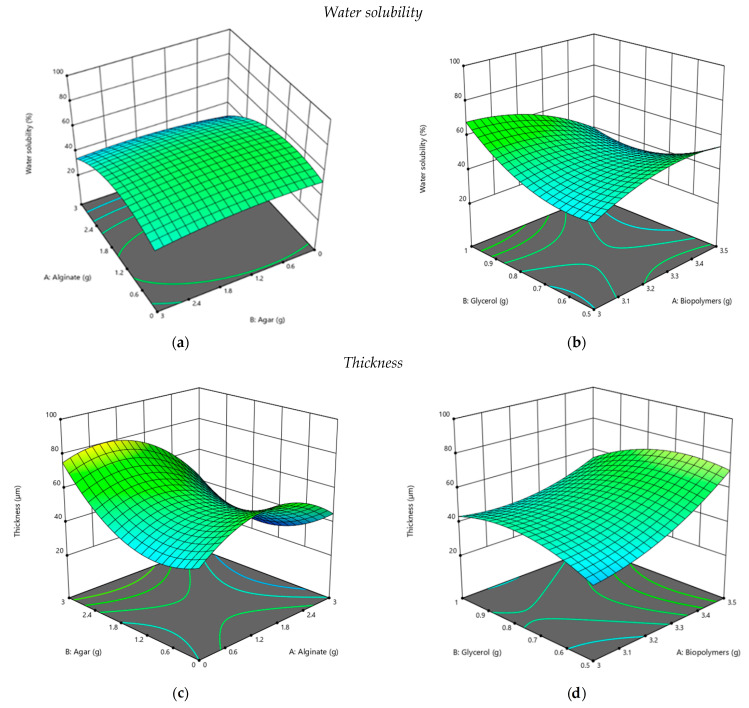

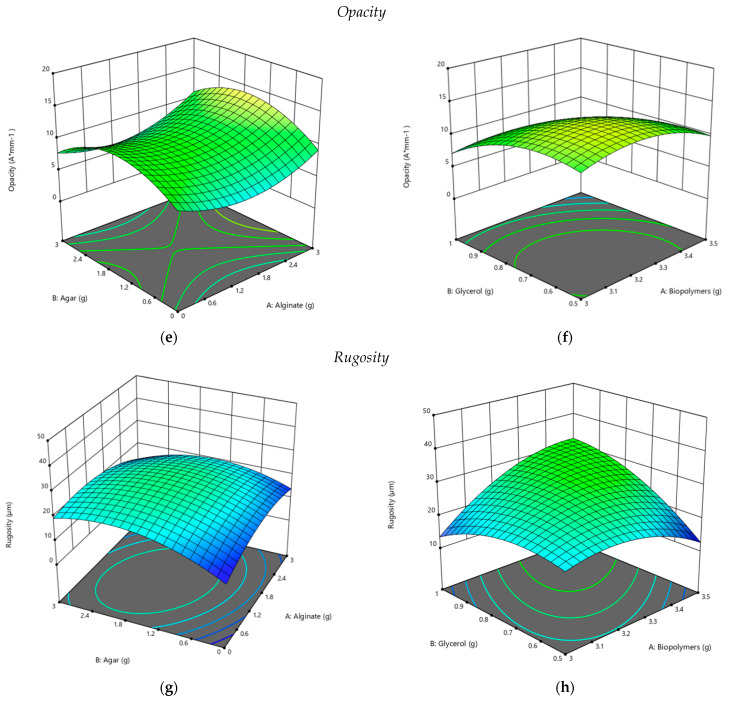
3 D Surface optimization of the films regarding the agar/alginate mass on water solubility (**a**), thickness (**c**), opacity (**e**), rugosity (**g**), and biopolymers-plasticizer mass on water solubility (**b**), thickness (**d**), opacity (**f**), and rugosity (**h**).

According to the images presented in Figure 6 and the statistical analysis, agar, alginate and glycerol can be used to obtain films that can be used as packaging materials in the food industry and beyond. The influence of their content on water solubility, thickness, opacity, and roughness was evaluated. Thus, for an average value of water solubility, the amounts of biopolymers are 1.24 g agar and 0.98 g alginate (Figure 6a), and the ratio of biopolymers:glycerol should be 3:1 (Figure 6b). The increase in solubility is directly proportional to the increase in alginate content, an aspect also found in the specialized literature [30,31,32]. Thickness is strongly influenced by the content in biopolymers. A higher agar content in the composition will facilitate the obtaining of materials with higher thickness, while the increase in the alginate mass will favor the development of thinner ones. According to the optimization, for the development of a thin film, it is necessary for the composition of biopolymers (agar:alginate) to maintain a ratio of 3:1.5 (Figure 6c). Similarly, increasing the glycerol content of the composition will favor the development of thinner films. To obtain medium-thick materials, the optimization results indicate a ratio of 3:0.5 biopolymer content:plasticizer (Figure 6d). The opacity is directly correlated with the addition of agar in the composition, as the films with a high addition of alginates are more transparent (Table 1, Figure 3). To obtain films with low opacity, the biopolymer:glycerol ratio must be maintained at 3.5:1 (Figure 6e). The increase in the mass of alginate and glycerol will favor the development of materials with low opacity and, implicitly, high transmittance (Figure 6f).

Depending on the use of the developed materials, the content in biopolymers can be modified, so as to finally obtain the product with the desired characteristics. In the case of packaging products with a higher content of lipids or compounds that can degrade in the presence of light, such as vitamins, the agar content of the composition will be higher, unlike that of alginate or plasticizer. If it is desired to obtain transparent foils, which better present the product contained, the amounts of alginate and glycerol in the composition of the material should be increased. The roughness of the materials is strongly influenced by the addition of biopolymers in the composition. In order to develop a material with low roughness, it is recommended to use biopolymers in a ratio of 3:1 agar:alginate (Figure 5g) and plasticizer in a ratio of 3.5:0.5 (Figure 6h).

## 5. Conclusions

The present study investigated the development and testing of new potential materials used for food packaging that require solubilization before consumption. The idea of obtaining such films arose in response to authorities’ concern about the intensive pollution of the environment due to conventional, oil-based packaging that is largely single-use and difficult to sort and recycle. The films were obtained from biopolymers, such as agar and alginate, being completely natural and edible. The sample with the most suitable characteristics was obtained from agar:alginate:glycerol in a ratio of 2:1:1. According to the tests performed, a higher amount of plasticizer in the composition was responsible for the improvement of the physical characteristics and solubility of the films. The results obtained indicate the possibility of using these foils as packaging material. To improve their properties, they can be enriched by the addition of natural compounds in the form of powders, purees, extracts, or essential oils, or in combination with other biopolymers, when their use is intended for packaging other categories of food. Although biopolymer films can be a substitute for plastics, there are still some issues that need to be addressed. First, since biopolymers are natural substances, influenced by the growing environment, climatic conditions, and extraction methods, there is the possibility that the reproducibility of the method does not ensure the development of the same product with identical characteristics, unlike synthetic materials. At the moment, production costs are higher than those for plastics, but as the demand and need for these biomaterials increase, costs can be mitigated. Besides this, the obtaining technology is easy and does not involve qualified personnel or special equipment.

## Figures and Tables

**Table 1:** The composition of biopolymer-based edible films.

Film	m_agar_ (wt %)	m_alginate_ (wt %)	m_glycerol_ (wt %)
**B1**	75.00	-	25.00
**B2**	-	75.00	25.00
**B3**	62.50	25.00	12.50
**B4**	50.00	37.50	12.50
**B5**	43.75	43.75	12.50
**B6**	37.50	50.00	12.50
**B7**	25.00	62.50	12.50
**B8**	40.87	34.13	25.00
**B9**	40.625	40.625	18.75
**B10**	50	43.75	18.75
**B11**	31.25	50	18.75
**B12**	31.25	43.75	18.75
**B13**	43.75	31.25	18.75
**B14**	50	25	25
**B15**	25	50	25
**B16**	37.5	37.5	25

The volume of water was 150 mL for each sample developed.

**Table 2:** The characteristics of the films after development.

Film	First Impression Evaluation									Observations
	Adhesivity	Surface	Flexibility	Multiple Bends	Margins Uniformity	Smell	Taste	Pores Fissures	Oral Solubility	
**B1**	low	rough	yes	yes	yes	no	sweet	no	low	combination with another biopolymers/increased plasticizer content
**B2**	high	smooth	medium	yes	yes	no	no	no	medium	thin foil, suitable as packaging material
**B3**	high	smooth	yes	yes	yes	no	no	no	very low	suitable for packaging products with high moisture content
**B4**	high	rough	no	no	yes	no	no	no	very low	tendency for exfoliation, brittle
**B5**	low	rough on the outside	medium	yes	yes	no	no	pores	low	unpleasant sensation during taste analysis
**B6**	medium	uneven	medium	yes	yes	no	sweet	pores	medium	slightly crunchy in the oral cavity, cannot be used in this form
**B7**	high	smooth	medium	yes	yes	no	no	pores	medium	the surface in contact with the foil is smooth, the outer has roughness
**B8**	high	uneven	medium	yes	yes	no	sweet	pores	medium	cannot be used in this form
**B9**	high	very smooth	yes	yes	yes	no	no	no	high	unpleasant sensation during taste analysis
**B10**	high	very smooth	yes	yes	yes	no	no	no	low, pleasant sensation	suitable as packaging material
**B11**	low	rough	no	no	yes	no	no	no	medium, unpleasant sensation	hard material, with a tendency to tighten, although it allows bending, cannot be used in this form
**B12**	medium	uneven	medium	yes	yes	no	no	no	medium	unpleasant sensation during taste analysis
**B13**	medium	very smooth	yes	yes	yes	no	sweet	no	medium, very pleasant sensation	crispy, pleasant sensation during taste analysis, good composition for packaging material
**B14**	medium	very smooth	yes	yes	yes	no	no	no	high	the most suitable composition for food supplements packaging material
**B15**	medium	very smooth	medium	yes	yes	no	sweet	no	medium, pleasant sensation	crispy, pleasant sensation during taste analysis, good composition for packaging material
**B16**	low	very smooth	yes	yes	yes	no	sweet	no	medium, pleasant sensation	crispy, pleasant sensation during taste analysis, good composition for packaging material

**Table 3:** Physical evaluation.

Film	Thickness (µm)	Retraction Ratio (%)	Color		
			L*	a*	b*
**B1**	70.00 ^a^ ± 0.91	7.49 ^c^ ± 0.25	86.22 ^b^ ± 0.88	−4.51 ^a^ ± 0.05	16.71 ^c^ ± 0.01
**B2**	43.25 ^h,i^ ± 0.83	42.84 ^a,b^ ± 0.64	91.89 ^a^ ± 0.27	−5.68 ^c,d^ ± 0.71	12.14 ^f^ ± 0.33
**B3**	50.75 ^d,e,f,g^ ± 0.64	32.93 ^a,b,c^ ± 0.30	90.62 ^a^ ± 0.33	−5.46 ^c,d^ ± 0.74	13.21 ^e,f^ ± 0.57
**B4**	66.75 ^a,b^ ± 0.68	11.78 ^c^ ± 0.81	90.46 ^a^ ± 0.27	−4.85 ^a,b^ ± 0.05	16.03 ^c^ ± 0.05
**B5**	36.50 ^i^ ± 0.64	51.76 ^a^ ± 0.36	90.81 ^a^ ± 0.24	−5.13 ^b,c^ ± 0.21	14.51 ^d^ ± 0.14
**B6**	60.75 ^b,c^ ± 0.14	19.71 ^b,c^ ± 0.71	90.41 ^a^ ± 0.32	−5.62 ^c,d^ ± 0.33	13.88 ^d,e^ ± 0.71
**B7**	44.75 ^f,g,h^ ± 0.83	40.86 ^a^ ± 0.01	91.06 ^a^ ± 0.71	−5.43 ^b,c^ ± 0.14	13.45 ^d,e^ ± 0.27
**B8**	54.50 ^c,d^ ± 0.51	27.97 ^a,b,c^ ± 0.46	91.03 ^a^ ± 0.33	−5.24 ^b,c^ ± 0.13	13.78 ^d,e^ ± 0.02
**B9**	55.60 ^c,d^ ± 0.87	26.52 ^a,b^ ± 0.76	92.09 ^a^ ± 4.45	−6.04 ^d,e^ ± 0.09	24.21 ^a^ ± 0.49
**B10**	45.20 ^h,i^ ± 0.61	43.83 ^a^ ± 0.32	92.77 ^a^ ± 0.16	−6.29 ^e^ ± 0.02	22.56 ^b^ ± 0.17
**B11**	46.25 ^e,f,g,h^ ± 0.68	38.87 ^a,b^ ± 0.22	92.60 ^a^ ± 0.34	−6.29 ^e^ ± 0.08	22.54 ^b^ ± 0.73
**B12**	44.75 ^f, g, h^ ± 0.09	40.86 ^a,b^ ± 0.71	92.27 ^a^ ± 0.15	−6.32 ± 0.02	23.85 ^a^ ± 0.26
**B13**	44.00 ^g,h^± 0.08	41.85 ^a^ ± 0.78	92.73 ^a^ ± 0.16	−6.43 ^e^ ± 0.02	23.19 ^a,b^ ± 0.21
**B14**	43.50 ^h,i^ ± 0.65	42.51 ^a,b^ ± 0.05	92.63 ^a^ ± 0.28	−6.37 ^e^ ± 0.03	22.62 ^b^ ± 0.14
**B15**	51.50 ^d,e,f^ ± 0.86	31.94 ^a,b,c^ ± 0.67	92.10 ^a^ ± 0.33	−6.42 ^e^ ± 0.07	23.72 ^a,b^ ± 0.75
**B16**	53.25 ^d,e^ ± 0.38	29.62 ^a,b,c^ ± 0.33	92.74 ^a^ ± 0.11	−6.44 ^e^ ± 0.02	23.17 ^a,b^ ± 0.17

Values (a–i) followed by the same letter are not significantly different (α: 0.05).

**Table 4:** Characteristics of the films.

Film	Moisture Content (%)	Water Solubility (%)	Water Activity Index a_w_
**B1**	18.86 ^b^ ± 0.64	40.71 ^h^ ± 0.41	0.417 ^e,f^ ± 0.04
**B2**	17.12 ^c^ ± 0.33	complete solubilization	0.427 ^d,e,f^ ± 0.33
**B3**	10.83 ^h^ ± 0.09	31.58 ^j^ ± 0.25	0.414 ^f^ ± 0.05
**B4**	10.26 ^i^ ± 0.61	39.63 ^i^ ± 0.33	0.437 ^c,d^ ± 0.66
**B5**	9.56 ^j^ ± 0.09	46.24 ^f^ ± 0.56	0.434 ^c,d^ ± 0.09
**B6**	14.17 ^f^ ± 0.65	40.24 ^h^ ± 0.66	0.418 ^e,f^ ± 0.33
**B7**	14.30 ^f^ ± 0.33	21.73 ^k^ ± 0.46	0.438 ^b,c,d^ ± 0.13
**B8**	14.55 ^f^ ± 0.07	43.00 ^g^ ± 0.32	0.436 ^c,d^ ± 0.66
**B9**	12.80 ^g^ ± 0.04	56.38 ± 0.06	0.429 ^d,e^ ± 0.90
**B10**	14.20 ^f^ ± 0.71	51.66 ^c^ ± 0.32	0.443 ^a,b,c^ ± 0.81
**B11**	13.25 ^g^ ± 0.48	62.08 d ± 0.30	0.418 ^e,f^ ± 0.72
**B12**	13.21 ^g^ ± 0.36	60.67 ^a^ ± 0.05	0.418 ^e,f^ ± 0.46
**B13**	15.85 ^e^ ± 0.05	52.75 ^a^ ± 0.71	0.451 ^a,b^ ± 0.33
**B14**	22.01 ^a^ ± 0.76	49.03 ^e^ ± 0.18	0.457 ^a^ ± 0.17
**B15**	16.50 ^d^ ± 0.31	57.52 ^b,c^ ± 0.33	0.427 ^d,e,f^ ± 0.98
**B16**	21.85 ^a^ ± 0.93	58.23 ^b^ ± 0.50	0.420 ^e,f^ ± 0.50

Values (a–k) followed by the same letter are not significantly different (α: 0.05).

## Data Availability

Data sharing not applicable.
